# Interaction between an *ADCY3* Genetic Variant and Two Weight-Lowering Diets Affecting Body Fatness and Body Composition Outcomes Depending on Macronutrient Distribution: A Randomized Trial

**DOI:** 10.3390/nu10060789

**Published:** 2018-06-19

**Authors:** Leticia Goni, Jose Ignacio Riezu-Boj, Fermín I. Milagro, Fernando J. Corrales, Lourdes Ortiz, Marta Cuervo, J. Alfredo Martínez

**Affiliations:** 1 Department of Nutrition, Food Sciences and Physiology, University of Navarra, 31008 Pamplona, Spain; lgoni@unav.es (L.G.); jiriezu@unav.es (J.I.R.-B.); fmilagro@unav.es (F.I.M.); jalfmtz@unav.es (J.A.M.); 2 Centre for Nutrition Research, University of Navarra, 31008 Pamplona, Spain; 3 Navarra Institute for Health Research (IdiSNA), 31008 Pamplona, Spain; 4 Biomedical Research Centre Network in Physiopathology of Obesity and Nutrition (CIBERobn), Institute of Health Carlos III, 28029 Madrid, Spain; 5 Centro Nacional de Biotecnología (CNB-CSIC), 28049 Madrid, Spain; fjcorrales@unav.es; 6 Biomedical Research Centre Network in Hepatic and Gastrointestinal diseases (CIBERehd), Institute of Health Carlos III, 28029 Madrid, Spain; 7 Genomic Core Facility, Center for Applied Medical Research (CIMA), University of Navarra, 31008 Pamplona, Spain; lortiz@unav.es; 8 Research Institute of Food & Health Sciences (IMDEA Food), 28049 Madrid, Spain

**Keywords:** *ADCY3*, gene–diet interaction, energy restricted diet, body fatness, body composition

## Abstract

The adenylate cyclase 3 (*ADCY3*) gene is involved in the regulation of several metabolic processes including the development and function of adipose tissue. The effects of the *ADCY3* rs10182181 genetic variant on changes in body composition depending on the macronutrient distribution intake after 16 weeks of the dietary intervention were tested. The *ADCY3* genetic variant was genotyped in 147 overweight or obese subjects, who were randomly assigned to one of the two diets varying in macronutrient content: a moderately-high-protein diet and a low-fat diet. Anthropometric and body composition measurements (DEXA scan) were recorded. Significant interactions between the *ADCY3* genotype and dietary intervention on changes in weight, waist circumference, and body composition were found after adjustment for covariates. Thus, in the moderately-high-protein diet group, the G allele was associated with a lower decrease of fat mass, trunk and android fat, and a greater decrease in lean mass. Conversely, in the low-fat diet group carrying the G allele was associated with a greater decrease in trunk, android, gynoid, and visceral fat. Subjects carrying the G allele of the rs10182181 polymorphism may benefit more in terms of weight loss and improvement of body composition measurements when undertaking a hypocaloric low-fat diet as compared to a moderately-high-protein diet.

## 1. Introduction

Obesity is a multifactorial disease in which various pathophysiological processes are involved, including the hormonal regulation of hunger and satiety, the activity of the central reward system, whole-body energy expenditure and the storage capacity for fat in the adipose tissue, and interactions with environmental factors [1]. In each of these metabolic functions a set of genes is implicated, with some of them being involved in a broad spectrum of processes [2]. The pleiotropic effect of such genes could be of special interest because they might explain some obesity causes.

Adenylate cyclase 3 (*ADCY3*), which encodes for a membrane-associated enzyme that catalyzes the formation of the secondary messenger cyclic adenosine monophosphate (cAMP) from adenosine triphosphate (ATP), could be a good candidate gene since it is widely expressed in most cell types and may be involved in a number of physiological and pathophysiological processes [3]. In animal studies, *ADCY3* expression has been found in certain regions of the brain, including the striatum and hypothalamus, and in the adipose tissue [4,5]. In humans, different genetic variants located near or in the *ADCY3* gene have been associated with obesity through candidate gene studies and genome-wide association studies (GWAS) [6,7,8,9,10,11,12]. Among the *ADCY3* genetic variants identified as related to obesity traits, the polymorphism rs10182181 has been replicated in the largest meta-analysis of GWAS on body mass index (BMI) carried out to date [12].

In this context, the aim of the present study was to analyze the effect of two different hypo-energetic diets with varying macronutrient distribution (moderately-high-protein diet vs. low-fat diet) on changes in anthropometric and body composition measurements after a 16-week dietary intervention according to the *ADCY3* rs10182181 genetic variant.

## 2. Materials and Methods

### 2.1. Study Population

The study encompassed a total of 147 overweight or obese subjects (BMI: 25–40 kg/m^2^) enrolled in a 16-week randomized clinical trial (clinical trial reg. no. NCT02737267, clinicaltrials.gov) of two hypo-energetic diets with different macronutrient composition, a low-fat diet and a moderately-high-protein diet (see Figure S1). Participants were recruited from October 2015 to February 2016 in the Metabolic Unit of the Centre for Nutrition Research of the University of Navarra. Major exclusion criteria included suffering from cardiovascular disease, type 1 diabetes, or type 2 diabetes treated with insulin; pregnant or lactating women; use of medications that affect body weight; weight change >3 kg within three months before the start of the intervention; unstable dose of medication for hyperlipidemia for type 2 diabetes patients; and treated with hypoglycemic and/or for hypertension.

The study protocol was approved by the Research Ethics Committee of the University of Navarra (ref. 132/2015). The research was performed in accordance with the ethical guidelines of the Declaration of Helsinki [13]. All participants provided written informed consent after they received an information sheet and additional verbal explanation of the protocol.

### 2.2. Diet Intervention

Energy requirements were individually evaluated from resting energy expenditure according to the Mifflin formula, multiplied for physical activity level calculated by a short 24 h physical activity questionnaire [14,15,16]. Diets presented the following target macronutrient composition: low-fat diet: 60% of total energy from carbohydrates, 18% from protein, and 22% from fat; and moderately-high-protein diet: 40% of total energy from carbohydrates, 30% from protein, and 30% from fat. Prescribed diets provided a 30% restriction of the total energy expenditure estimated for each subject. No initial prescribed diets had less than 1200 kcal/day.

Subjects were randomly assigned to one of the two diets by a specific logarithm design for the study by MATLAB using stratified block randomization according to gender, age groups (<45 years and ≥45 years), ethnicity (Caucasian and Hispanic) and BMI (overweight, BMI 25–29.9 kg/m^2^; and obesity, BMI 30–40 kg/m^2^).

Compliance analysis to the recommended diet of the participants was conducted taking into account a three-day-weighed food record (two weekdays and one weekend day) at two times: the eighth week and at the end of the intervention period (the 16th week). Total energy intake and nutrient content were determined using validated Spanish food composition tables and appropriate software [17,18,19].

### 2.3. General Measurements

Anthropometric and body composition determinants were taken at the beginning and at the end of the study in fasting conditions with the subjects in their underwear, as described elsewhere [20]. Body weight was assessed using a Tanita BC-418 scale (Tanita, Tokyo, Japan) and height was measured using a wall-mounted stadiometer. BMI was calculated by dividing weight (kg) by the square of height (m). Waist circumference (WC) was measured using a stretchable tape measure, midway between the lower margin of the least rib and the top of the iliac crest or according to the circumference at the level of the umbilicus if it was not possible to identify the least rib or the iliac crest. Body composition and distribution (fat mass, lean mass, trunk fat, android fat, gynoid fat, and visceral fat) was analyzed by dual energy x-ray absorptiometry (DEXA) scan (DEXA Lunar Prodigy, GE Medical Systems, Madison, WI, USA). Baseline dietary intake was analyzed by a previously validated 137-item food frequency questionnaire [21,22,23].

### 2.4. Genotyping

For genotyping, epithelial buccal cells were collected using a liquid-based kit (ORAcollect-DNA, OCR-100, DNA Genotek, Ottawa, ON, Canada). Genomic DNA was extracted with a Maxwell 16 Buccal Swab LEV DNA Purification Kit in the Maxwell 16 instrument (Promega, Madison, WI, USA). *ADCY3* rs10182181 was genotyped by next-generation sequencing using a pre-designed SNP panel (Ion AmpliSeq Custom NGS DNA Panels, Thermo Fisher Scientific Inc., Waltham, MA, USA), which was validated in the Ion Torrent PGM (Thermo Fisher Scientific Inc.). Data were analyzed with the Torrent Variant Caller plugin for the Ion Torrent Sequencing platform and R software.

### 2.5. Statistical Analyses

The primary end points of the present study were changes in anthropometric (weight, waist circumference) and body composition (fat mass, lean mass, trunk fat, android fat, gynoid fat, and visceral fat). General linear models for continuous variables and the chi-squared test for categorical variables were applied for the comparison according to genotype groups at baseline. Chi-squared tests were also used to assess the Hardy–Weinberg equilibrium (HWE). The adherence to the diet across the genotype groups was examined using general linear models adjusted for age and gender. Multivariate general linear models were applied to test changes in primary outcomes according to genotype groups after adjustment for covariates (model 1: age, gender and the respective baseline variable; and model 2: model 1 plus BMI at baseline). The interaction term (e.g., *ADCY3* genotype x diet) was included in the models in order to test potential gene–diet interactions. The polymorphism was analyzed for additive and co-dominant effects. The study had 80% power, assuming a mean difference in the primary outcome (weight loss) of 2 kg and a standard deviation of 3.5 kg at a significance level of 0.05. For the statistical analysis, STATA/SE version 12.0 (StataCorp, Collegue Station, TX, USA) was used. The statistical significance was considered at *p* < 0.05.

## 3. Results

Baseline characteristics were similar among participants assigned to the moderately-high-protein diet and the low-fat diet (Table 1). Forty (27%) subjects failed to complete the dietary intervention with no significant differences (*p* = 0.83) among diets (see Figure S1). The changes in body fatness and body composition measurements were statistically significant in both diets (all *p* values < 0.001). The mean weight loss was 7.6 kg in the moderately- high-protein diet, and 8.1 kg in the low-fat diet, with no group difference (*p* = 0.62). There were no differences either in the changes in BMI and WC as well as in any of the assessed body composition measurements (fat mass, lean mass, trunk fat, android fat, gynoid fat, and visceral fat) (Table 1).

The minor allele frequency (G allele) of the *ADCY3* rs10182181 genetic variant was 0.51 among the study participants. The polymorphism was in Hardy–Weinberg Equilibrium (HWE) (*p* > 0.05). Baseline characteristics of the participants included in the present study were also analyzed according to the *ADCY3* rs10182181 genetic variant (Table 2). The distribution of gender and diet group did not differ depending on the genotype. No significant difference was found in dietary intake or in anthropometric and body composition measurements at baseline examination, whereas a significant difference was observed for age (*p* = 0.007). Those subjects carrying the G allele presented a lower average age than homozygous subjects for the A allele.

In the moderately-high-protein diet group, the targets of macronutrient intakes during the intervention were achieved (Table 3). However, the targets of macronutrient intake were not fully achieved among subjects of the low-fat diet. On the other hand, the reported dietary intake confirmed that participants modified their intakes of macronutrients in the direction of the intervention and significant differences were observed for the intake of fat, protein and carbohydrates between groups (all *p* values < 0.001). There were no significant differences in energy and macronutrient intakes depending on the *ADCY3* rs10182181 genotype and group diet (Table 3).

**Figure 1:**
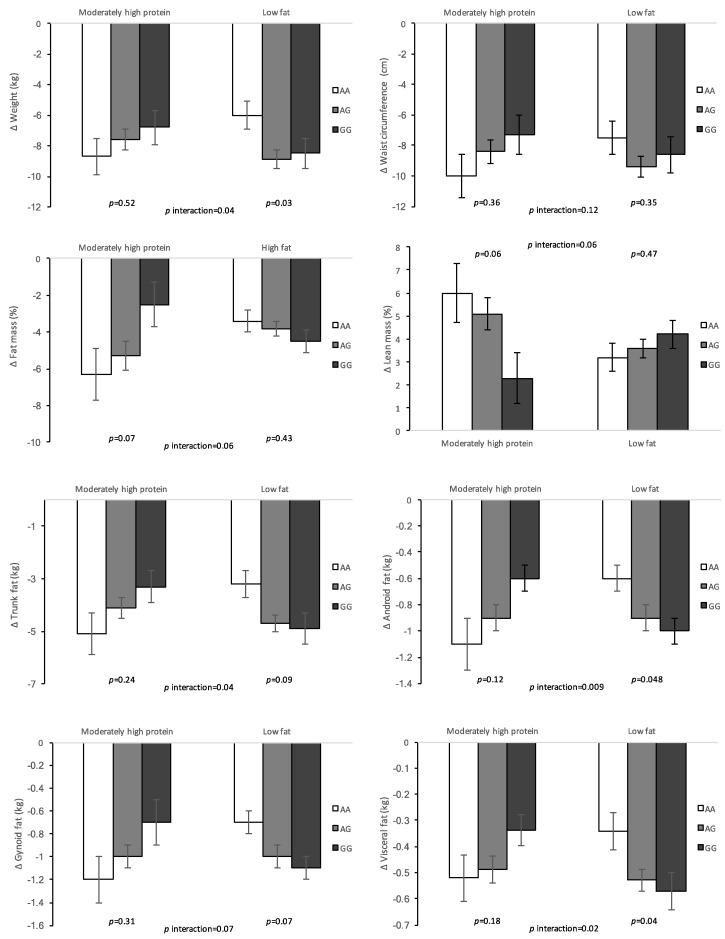
Effect of the *ADCY3* rs10182181 genetic variant on changes in body fatness and composition in response to moderately-high-protein/low-fat diet after 16 weeks of diet intervention (co-dominant model).

In the present study, there were no significant associations of the *ADCY3* polymorphism with changes in anthropometric and body composition measurements during the 16-week dietary intervention after adjustment for age, sex, the baseline value for the respective outcome, BMI at baseline and dietary group (data not shown). However, we found significant interactions between *ADCY3* rs10182181 genotype and dietary intake on changes in body fatness and body composition measurements (Table 4). Specifically, the rs10182181 genetic variant interacted with dietary intake on changes in weight, WC, fat mass, percentage of fat mass, percentage of lean mass, trunk fat, android fat, gynoid fat, and visceral fat (model 2 all *p* for interaction < 0.05) (Table 4). After adjusted for age, gender, the respective baseline variable and BMI at baseline the G allele was significantly associated with a lower decrease of fat mass, trunk fat and android fat, and a greater decrease of lean mass in the moderately-high protein diet group (*p* = 0.01, *p* = 0.03, *p* = 0.01 and *p* = 0.01, respectively). Conversely, carrying the G allele was associated with a greater decrease in trunk fat, android fat, gynoid fat, and visceral fat when consuming a low-fat diet, once adjusted for covariates (*p* = 0.04, *p* = 0.02, *p* = 0.03 and *p* = 0.02, respectively). Similar trends were found for the co-dominant model (Figure 1).

## 4. Discussion

The current study reported for the first time a significant gene–diet interaction between *ADCY3* rs10182181 genetic variant and dietary macronutrient composition of low calorie diets on changes in anthropometric and body composition measurements. Among individuals with the rs10182181 G-allele consuming the low-fat diet showed greater effect on changes in trunk fat, android fat and gynoid fat, compared with the moderately-high-protein diet over the 16-week dietary intervention.

The *ADCY3* gene encodes for an enzyme that converts the ATP to cAMP, which is a second messenger used for intracellular signal transduction [3]. This messenger is involved in a large number of physiological metabolic processes including the regulation of carbohydrate and lipid metabolism, and the development and function of the adipose tissue regulating the expression of genes involved in adipogenesis, thermogenesis and lipolysis [3,24]. In 2008, for the first time Nordman et al. reported that the *ADCY3* rs2033655 and rs1968482 genetic variants were related to obesity but not to type 2 diabetes in a Swedish male population [6]. Subsequently, the authors replicated the genetic association in a large cohort of Chinese adults [7]. In this study, the genetic variants rs1127568, rs7604576 and rs753529 were significantly associated with obesity. Moreover, several GWAS have identified *ADCY3* as a gene associated with obesity in adult and children populations [6,7,8,9,10,11,12]. For example, the largest meta-analysis of GWAS on BMI carried out-to-date found that the *ADCY3* rs10182181 and rs713586 variants were associated with BMI [12]. In another GWAS the rs713586 polymorphism, which is in strong linkage disequilibrium with the rs10182181 polymorphism, was identified as an associated BMI variant as well as an expression quantitative trait loci (eQTL) since it was related to *ADCY3* gene expression in different tissues (lymphocytes, omental fat and blood) [8,25]. Interestingly, after an in silico analysis we have confirmed that the rs10182181 polymorphism is in the promoter region of the *ADCY3* gene. Particularly, it is located in a binding site for transcription factors including USF1, POLR2A, BHLHE40, JUNB, and CREM. This observation suggests that rs10182181 polymorphism may be important for the biological function of the *ADCY3* gene.

In accordance with the studies in humans, the *Adcy3* knockout mice (*Adcy3*^−/−^) developed obesity characterized by an increase in fat mass and larger adipocytes [26]. Furthermore, the authors reported that *Adcy3*^−/−^ mice exhibited reduced physical activity, increased food intake, and leptin insensitivity; and speculated that these phenotypic changes could be associated with disruption of cAMP signaling in primary cilia of the hypothalamus. Recently, the same group using a floxed *Adcy3* mouse strain determined that Adcy3 in the hypothalamus regulated energy expenditure [27]. Apart from the hypothalamus, it has been found that Adcy3 is overexpressed in pancreatic islets of non-obese-type 2 diabetic Goto–Kakizaki rats, playing an important role in insulin secretion regulation [5]. There is also some evidence to suggest that Adcy3 may play specific physiological roles in major depression and sleep disruption, which are disorders strongly associated with the obesity phenotype [28]. Moreover, Adcy3 may functionally couple to melanocortin 4 receptor (Mc4r) in the hypothalamus, because activation of adenylyl cyclase activity by alpha-melanocyte stimulating hormone downstream in the leptin pathway is required for the anorectic activity of leptin [29]. In fact, *Mc4r* and *Adcy3* knockout mice exhibit similar phenotypes, including obesity and hyperinsulinemia [26,30].

In the present study, macronutrient distribution significantly modified the effect of the *ADCY3* genetic variants on changes in anthropometric and body composition measurements. The participants with the G allele of the rs10182181 *ADCY3* variant showed a greater decreased in fat mass, trunk fat, android fat, gynoid fat, and visceral fat when consuming a low-fat diet. In this sense, it has been reported that a high fat diet decreased the Adcy3 expression in white adipose tissue, liver and muscle [31]. This haploinsufficiency confers decreased expression of genes involved in thermogenesis, fatty acid oxidation and insulin signaling in mice and conversely, it enhanced the expression of genes related to adipogenesis in peripheral tissues. Moreover, mice with a gain-of-function mutation in Adcy3 presented increased Adcy3 activity and cAMP production and consequently the mutation protects mice from high fat diet-induced metabolic disorders [32]. However, the mechanisms underlying the modulation of macronutrient intake on the *ADCY3* genetic variant are not fully understood and further experimental studies are needed.

To our knowledge, this is the first investigation to analyze the effect of the *ADCY3* genetic variant on changes in fatness and body composition in response to two weight loss diets with different macronutrient composition. However, several limitations should be considered. First, analyzing the allocated diet (moderately high-protein vs. low-fat diet) rather than actual diet may have obscured the interactions found in this study. Nevertheless, using reported intake has its own problems with potential misreporting and would challenge the advantage of the study’s randomized design. Second, it is difficult to determine which macronutrient plays the key role behind the observed interactions because both weight loss diets differed in the content of carbohydrates, protein and fat. Third, the participants of the study were of self-reported European ancestry. Thus, it is unknown whether our results can be generalized to other ethnic groups.

In conclusion, carriers of the minor allele of *ADCY3* genotypes might have a better response to a weight-loss dietary intervention by choosing a low-fat diet than a moderately-high-protein diet. Identifying gene–diet interactions on response to metabolic features may assist therapists in assigning more personalized and successful treatments, which could improve long-term weight management [33].

## Figures and Tables

**Table 1:** Characteristics at baseline and after 16 weeks of intervention concerning anthropometric and body composition measurements.

		Moderately-High-Protein		Low-Fat		*p* ^a^	*p* ^b^
		Baseline (*n* = 72)	Change (*n* = 53)	Baseline (*n* = 75)	Change (*n* = 54)		
Age (years)		45.6 (11.2)	-	47.5 (8.6)	-	0.28	-
Female sex		48 (66.7)	-	51 (68.0)	-	0.87	-
Body weight (kg) ^c^		88.5 (13.2)	−7.6 (4.0) ^d^	88.8 (13.3)	−8.1 (4.1) ^d^	0.76	0.62
BMI (kg/m^2^) ^c^		31.7 (3.6)	−2.7 (1.4) ^d^	32.1 (3.8)	−2.9 (1.3) ^d^	0.56	0.71
WC (cm) ^c^		103.5 (11.2)	−8.4 (4.4) ^d^	103.8 (10.6)	−8.8 (4.5) ^d^	0.99	0.73
Body composition							
	Fat mass (%) ^c^	42.6 (9.1)	−4.8 (5.3) ^d^	41.9 (6.9)	−3.9 (2.5) ^d^	0.37	0.13
	Lean mass (%) ^c^	54.2 (9.2)	4.6 (5.4) ^d^	55.0 (6.6)	3.6 (2.4) ^d^	0.34	0.12
	Trunk fat (kg) ^c^	20.3 (5.0)	−4.1 (2.5) ^d^	20.6 (4.7)	−4.3 (2.4) ^d^	0.81	0.87
	Android fat (kg) ^c^	3.6 (1.0)	−0.8 (0.5) ^d^	3.6 (0.9)	−0.9 (0.5) ^d^	0.78	0.98
	Gynoid fat (kg) ^c^	5.9 (1.5)	−0.9 (0.7) ^d^	6.0 (1.7)	−1.0 (0.5) ^d^	0.74	0.84
	Visceral fat (kg) ^c^	1.5 (0.9)	−0.5 (0.4) ^d^	1.7 (0.9) ^d^	−0.5 (0.4) ^d^	0.37	0.92

BMI, Body mass index, WC, Waist circumference; Data are expressed as mean (SD) or *n* (%); ^a^ Comparison of baseline characteristic by dietary group; ^b^ Comparison of changes in anthropometric and body composition measurements by dietary group; ^c^ Adjusted for age and gender; ^d^ Significant differences in changes in anthropometric and body composition measurements in each group.

**Table 2:** Baseline characteristics concerning dietary and body composition measurements of the participants, depending on the *ADCY3* rs10182181 genetic variant.

		AA (*n* = 35)	AG (*n* = 74)	GG (*n* = 38)	*p*
Age (years)		50.9 (9.0)	46.0 (9.5)	43.8 (10.7)	0.007
Sex					0.16
	Male	11 (31.4)	20 (27.0)	17 (44.7)	
	Female	24 (68.6)	54 (73.0)	21 (55.3)	
Diet group					0.84
	Moderately-high-protein	16 (45.7)	36 (48.7)	20 (52.6)	
	Low-fat	19 (54.3)	38 (51.3)	18 (47.4)	
Dietary intake per day					
	Energy (kcal)	2907 (849)	3023 (855)	3038 (1001)	0.78
	Protein (%)	16.7 (3.4)	16.5 (2.5)	17.1 (3.1)	0.55
	Fat (%)	38.8 (6.2)	39.6 (6.0)	40.0 (5.2)	0.67
	Carbohydrate (%)	42.8 (8.5)	41.9 (6.5)	40.8 (7.0)	0.52
Body weight (kg)		88.2 (13.1)	87.6 (12.6)	91.0 (14.6)	0.43
BMI (kg/m^2^)		32.7 (3.9)	31.6 (3.5)	31.9 (3.6)	0.34
WC (cm)		105.9 (10.2)	102.2 (10.5)	104.5 (12.0)	0.22
Body composition ^a^					
	Fat mass (%)	42.1 (5.4)	42.2 (8.9)	42.4 (8.5)	0.99
	Lean mass (%)	54.8 (5.1)	54.7 (8.7)	54.4 (8.5)	0.98
	Trunk fat (kg)	20.7 (3.9)	19.9 (4.8)	21.2 (5.7)	0.40
	Android fat (kg)	3.6 (0.8)	3.5 (0.9)	3.8 (1.2)	0.30
	Gynoid fat (kg)	5.8 (1.5)	6.0 (1.7)	6.0 (1.6)	0.87
	Visceral fat (kg)	1.7 (0.9)	1.5 (0.9)	1.7 (1.0)	0.36

BMI, Body mass index; WC, Waist circumference; Data are expressed as mean (SD) or *n* (%); ^a^ Data available for 146 participants (AA = 34, AG = 74, GG = 38).

**Table 3:** Dietary intake by the *ADCY3* rs10182181 genetic variant and diet group during the intervention.

		All Population	AA	AG	GG	*p*
Moderately-high protein diet ^a^						
	Energy (kcal)	1340 (286)	1297 (180)	1294 (238)	1490 (414)	0.11
	Protein (%)	28.9 (4.3)	29.2 (3.5)	29.7 (4.2)	26.7 (4.4)	0.12
	Fat (%)	30.8 (5.0)	31.7 (5.4)	30.1 (4.9)	32.1 (5.1)	0.43
	Carbohydrate (%)	42.5 (4.3)	42.1 (3.7)	41.9 (3.8)	44.2 (5.7)	0.27
Low-fat diet ^b^						
	Energy (kcal)	1324 (236) ^c^	1313 (274)	1291 (231)	1429 (185)	0.29
	Protein (%)	22.3 (3.7) ^d^	22.0 (4.2)	22.2 (3.2)	22.8 (4.8)	0.89
	Fat (%)	27.0 (5.7) ^d^	25.6 (8.3)	27.4 (3.7)	27.6 (6.6)	0.61
	Carbohydrate (%)	53.3 (7.1) ^d^	54.5 (8.5)	53.1 (6.5)	52.1 (7.5)	0.72

^a^ Data were available for 52 individuals (AA *n* = 10, AG *n* = 30, GG *n* = 12); ^b^ Data were available for 51 individuals (AA *n* = 13, AG *n* = 28, GG *n* = 10); ^c^ *p* = 0.76 when it was compared with moderately-high-protein diet; ^d^ *p* < 0.001 when it was compared with moderately-high-protein diet.

**Table 4:** Effect of the *ADCY3* rs10182181 genetic variant on changes in body fatness and composition in response to moderately-high-protein/low-fat diet after 16 weeks of diet intervention (additive model).

		Moderately-High-Protein		Low-Fat		*p* Interaction
		β (SE)	*p*	β (SE)	*p*	
**Model 1**						
	Δ Weight (kg)	0.95 (0.82)	0.25	−1.32 (0.69)	0.06	0.02
	Δ WC (cm)	1.20 (0.95)	0.21	−0.88 (0.84)	0.30	0.06
	Δ Fat mass (kg)	1.26 (0.76)	0.10	−0.95 (0.57)	0.10	0.01
	Δ Fat mass (%)	1.65 (0.92)	0.08	−0.54 (0.44)	0.22	0.03
	Δ Lean mass (kg)	−0.54 (0.96)	0.57	−0.15 (0.30)	0.60	0.58
	Δ Lean mass (%)	−1.61 (0.90)	0.08	0.48 (0.42)	0.25	0.04
	Δ Trunk fat (kg)	0.89 (0.50)	0.08	−0.72 (0.41)	0.08	0.008
	Δ Android fat (kg)	0.22 (0.11)	0.049	−0.18 (0.08)	0.02	0.002
	Δ Gynoid fat (kg)	0.18 (0.14)	0.20	−0.19 (0.08)	0.03	0.02
	Δ Visceral fat (kg)	0.08 (0.06)	0.16	−0.12 (0.05)	0.02	0.005
**Model 2**						
	Δ WC (cm)	1.72 (0.94)	0.07	−0.61 (0.81)	0.45	0.03
	Δ Fat mass (kg)	1.49 (0.78)	0.06	−1.08 (0.57)	0.06	0.006
	Δ Fat mass (%)	2.21 (0.89)	0.01	−0.57 (0.43)	0.20	0.01
	Δ Lean mass (kg)	−0.41 (0.92)	0.65	−0.15 (0.30)	0.63	0.52
	Δ Lean mass (%)	−2.13 (0.84)	0.01	0.50 (0.41)	0.23	0.01
	Δ Trunk fat (kg)	1.15 (0.52)	0.03	−0.85 (0.40)	0.04	0.006
	Δ Android fat (kg)	0.28 (0.11)	0.01	−0.19 (0.08)	0.02	0.001
	Δ Gynoid fat (kg)	0.27 (0.14)	0.05	−0.20 (0.09)	0.03	0.008
	Δ Visceral fat (kg)	0.10 (0.06)	0.09	−0.11 (0.05)	0.02	0.005

WC, Waist circumference; β represents changes in outcomes for the increasing number of G allele of the *ADCY3* rs10182181 variant; Model 1: Adjusted for age, gender, and the respective baseline variable; Model 2: Model 1 plus BMI at baseline.

## Supplementary Materials

The following are available online at http://www.mdpi.com/2072-6643/10/6/789/s1. Figure S1: Flow-chart.
