# Supplementary figures and images for: Interaction between an ADCY3 Genetic Variant and Two Weight-Lowering Diets Affecting Body Fatness and Body Composition Outcomes Depending on Macronutrient Distribution: A Randomized Trial
Source: Nutrients. 2018 Jun 19;10(6):789. doi: 10.3390/nu10060789 (PMC6024801; doi:10.3390/nu10060789)

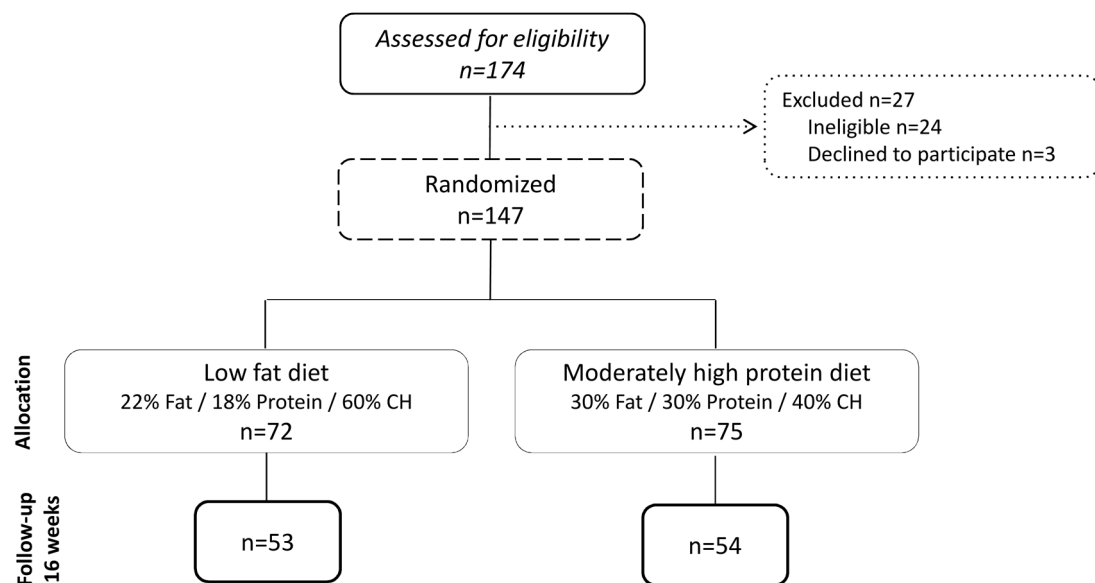

**Supplemental Figure 1. Flow-chart**

Supplement: Supplementary file 1 [file nutrients-10-00789-s001.pdf]
